# Sustainable Bisphenol A Alternatives from Vanillin and Erythritol Using Zeolite Catalysts

**DOI:** 10.1002/cssc.202500923

**Published:** 2025-07-31

**Authors:** Kevin M. Sabel, Joby Sebastian, Regina Palkovits

**Affiliations:** ^1^ Chair of Heterogeneous Catalysis and Technical Chemistry Institute for Technical and Macromolecular Chemistry RWTH Aachen University Worringerweg 2 52074 Aachen Germany; ^2^ Institute for a Sustainable Hydrogen Economy Forschungszentrum Jülich GmbH Marie‐Curie‐Str. 5 52428 Jülich Germany; ^3^ Max‐Plank‐Institut for Chemical Energy Conversion Stiftstr. 34‐36 45470 Mülheim an der Ruhr Germany

**Keywords:** acetalization, bisphenol derivates, erythritol, vanillin, zeolites

## Abstract

Bisphenol A (BPA), a key monomer used in polymer production, poses significant health and environmental risks, necessitating the need for sustainable alternatives. This study establishes a sustainable route for BPA alternatives via the acetalization of biomass‐derived vanillin and erythritol using industrially relevant zeolite catalysts. Among various homogeneous and heterogeneous Brønsted and Lewis acid catalysts tested, heterogeneous mordenite zeolite (SiO_2_/Al_2_O_3_ = 40) achieves the highest BPA derivative yield (90%). Kinetic studies confirm a consecutive reaction mechanism, with the hemiacetal‐to‐acetal step being the rate‐determining step. The catalyst is recyclable and maintains stable performance over five consecutive runs. Detailed structural characterizations, reactant accessibility studies, conditional experiments, and probe molecule tests reveal that a higher external surface area, Brønsted acid sites on the external surface, and greater hydrophobicity are key to the zeolite's high efficiency. These features facilitate the adsorption and desorption of large reactant and product molecules, circumventing pore‐diffusion limitations while evading byproduct water‐induced deactivation. This study not only demonstrates the use of industrial zeolites for sustainable BPA derivatives from biorenewables but also highlights critical structural and compositional parameters for optimizing catalyst design. Also, the findings provide a framework for rationalizing zeolite‐based catalysts for a broad range of biobased BPA alternatives.

## Graphical abstract

This study investigates a sustainable alernative to Bisphenol A synthesized via acetalization of vanillin and erythritol using zeolite catalysts. Among the tested zeolites, mordenite (SiO_2_/Al_2_O_3_ = 40) exhibited the highest yield of 90%. The catalyst efficiency depends on the accessibility of external Bronsted acid sites. The catalyst demonstrated excellent recyclability, highlighting its potential use in sustainable industrial Bisphenol A alternatives.

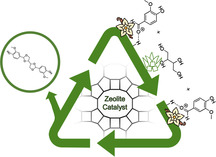

## 1 Introduction

Bisphenol A (BPA) with its readily accessible aromatic OH groups serves as a monomer ideal for the production of various polymer materials, including polyesters, polycarbonates, and epoxy resins.^[^ ^1^, ^2^, ^3^ ^]^ The global annual production of BPA is ≈9.2 million metric tons as of 2023, driven by high demand in the polycarbonate and epoxy resin sectors. Production is projected to grow at a compound annual growth rate (CAGR) of 3% through 2032. Despite its versatility, there are major concerns about the use of BPA containing plastics that come into contact with food, for instance, food packaging.^[^ ^4^ ^]^ This is because BPA can accumulate in the human body and have serious consequences for human health, such as fertility issues, diabetes, obesity, or cardiovascular problems.^[^ ^5^ ^]^ In view of these health risks, many countries have begun to regulate or ban the use of BPA in food packaging and other products that come into contact with food like kitchen utensils. The European Union, for example, has set strict limits on the migration of BPA from food contact materials. In 2015, the tolerable daily intake (TDI) of BPA in food was 4 μg kg^−1^ body weight and was drastically reduced to 0.2 ng kg^−1^ in 2023.^[^ ^6^ ^]^ Some countries, such as France, have completely banned BPA in baby bottles and other baby products. Ongoing research is focused on finding safer alternatives to BPA and gaining a better understanding of the long‐term effects of exposure to BPA and its substitutes.^[^ ^7^ ^]^

BPA is industrially produced via condensation of phenol and acetone, using an acidic catalyst. Phenol is typically obtained via cumene hydroperoxide synthesis, while acetone is the byproduct of the same synthesis or is derived from the oxidation of propene.^[^ ^8^ ^]^ Currently, the study of biomass‐derived bisphenols is of great interest to both the scientific community and the chemical industry, as they provide a promising foundation for the production of sustainable polymers.^[^ ^9^, ^10^, ^11^ ^]^ In particular, lignin‐based substrates like vanillin offer a viable alternative to toxic and fossil‐based chemicals like terephthalates, parabens, and phenol derivates.^[^ ^12^, ^13^, ^14^, ^15^ ^]^ Bisphenols can be synthesized by the acetalization of vanillin with pentaerythritol.^[^ ^9^, ^16^ ^]^ Acetalization reactions are acid catalyzed and involve an aldehyde or ketone reacting with two equivalents of an alcohol. This condensation results in the formation of the acetal and water as a byproduct. Since the reaction is reversible, it is essential to remove the byproduct water to drive the equilibrium towards acetal formation. Using a clay mineral as an acidic catalyst and a Dean–Stark apparatus for water removal, almost 95% yield to the corresponding acetal from vanillin and pentaerythritol has been reported.^[^ ^16^ ^]^ However, the process has certain disadvantages, such as the nonsustainable, fossil‐based synthesis of pentaerythritol and the near stoichiometric use of the clay catalyst (60 wt% relative to vanillin). Further research is essential to identify a biomass‐derived, sustainable feedstock alternative to pentaerythritol, as well as a catalyst that operates effectively even when used in small quantities. A promising alternate to pentaerythritol is bioderived erythritol, which can be obtained via fermentation of glucose.^[^ ^17^ ^]^ This could enable the production of fully biomass‐based bisphenols.

Zeolites are widely acclaimed for their pivotal role as solid acid catalysts in many industrial processes.^[^ ^18^, ^19^ ^]^ They possess Brønsted acidity, which can be modulated by adjusting the SiO_2_/Al_2_O_3_ ratio. Acetalization reactions can be successfully performed with zeolite catalysts.^[^ ^18^, ^19^ ^]^ Due to their ordered and well‐defined crystalline structure, pore size distribution, and the ability to finely tune their acidity and acid density for specific reactions, zeolites are expected to be superior to clay catalysts. Furthermore zeolites such as mordenite (MOR) have already found application in industrial processes, underlining their practical relevance and robustness under process conditions, with an annual market size of USD 1.2 Billion (2024).^[^ ^20^ ^]^

**Scheme 1:**
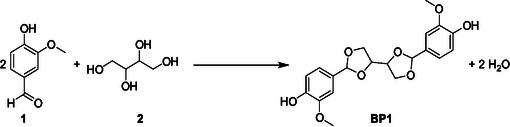
Reaction pathway for the acetalization of vanillin (1) with erythritol (2) to produce BP1.

This study investigates the synthesis of a biobased BPA alternative through the acetalization of vanillin and erythritol (**Scheme** **1**) using various industrially employed zeolite catalysts (Hβ, ZSM5, HY, and MOR). A comprehensive screening of both homogeneous and heterogeneous catalysts, with a particular focus on various zeolites, was conducted to identify the most effective zeolite catalyst for the reaction. Structure‐activity and reactant accessibility relationships were explored to correlate the zeolite's acidic and textural properties to BPA yield. Additionally, the stability of the most efficient zeolite was evaluated through recyclability studies and postcatalyst characterizations. The findings in this study provide valuable insights for the rational design of zeolite‐based catalysts in the development of sustainable BPA alternatives.

## 2 Results and Discussion

### 2.1 Screening of Different Solid/Liquid Acid Catalysts

For the initial screening, various heterogeneous and homogeneous catalysts were evaluated. Established acids for acetalizations are commonly used homogeneous acid catalysts including *p*‐toluene sulfonic acid (*p*TSA) or sulfuric acid. However, the need for neutralization and separation results in ecological and economic challenges. To circumvent these challenges, heterogeneous acid catalysts, such as commercially used zeolites, are tested for the reaction. The aim is to find the most suitable and efficient catalyst in terms of conversion and product yield (BP1). Additionally, factors such as product recovery and catalyst separation are also taken into account during the initial screening study. **Table** **1** summarizes the various catalysts employed and their corresponding catalytic performance in BP1 synthesis.

**Table 1:** Catalyst screening for the acetalization of vanillin (1) with erythritol (2) to the corresponding bisphenol derivate BP1.

Entry	Catalyst	*X* a) [%]	*Y* a) [%]	*S* a) [%]
1	No catalyst	0	0	0
2	Montmorillonite K‐10	0	0	0
3	HCZB‐25	90	45	50
4	HCZB‐30	72	22	30
5	HCZB‐150	79	5	6
6	HZSM5‐30	81	36	44
7	HZSM5‐50	38	9	24
8	HZSM5‐80	45	9	20
9	HY‐5.1	20	8	40
10	HY‐60	81	66	81
11	HCZM‐14	90	53	58
12	HCZM‐20.6	76	43	56
13	HCZM‐40	99	90	91
14	AmberLyst‐15	95	68	72
15	AmberLyst‐36	65	36	55
16	H_2_SO_4_ b)	0	0	0
17	*p*TSAc)	91	78	86
18	Al_2_O_3_	0	0	0
19	Sc(SO_3_CF_3_)_3_	84	0	0

Reaction conditions: *n*(vanillin) = 3 mmol, *n*(erythritol) = 1.8 mmol, catalyst amount = 10 wt% (w.r.t. vanillin), 25 mL toluene, *T *= 130 °C, *t *= 4 h, air stream.

a) Conversion (*X*) of vanillin, yield (*Y)* of BP1, and selectivity (*S*) of BP1 determined by ^1^H‐NMR spectroscopy using mesitylene as the internal standard.

b) 0.05 mmol H_2_SO_4_.

c) 0.05 mmol pTSA.

First, the reaction without the addition of any catalyst was carried out as a blind test for comparison. No conversion of the reactants was observed (Table 1, entry 1). Over Montmorillonite K‐10 catalyst, also no conversion of reactants to BP1 was detected (Table 1, entry 2). This may be attributed to the significantly lower catalyst loading of 10 wt%, compared to the 60 wt% reported in the previous study.^[^ ^16^ ^]^ Regarding the Hβ‐zeolites (HCZB) with a SiO_2_/Al_2_O_3_‐ratio of 25 (HCZB‐25), a moderate yield of 45% to BP1 was achieved at 90% conversion of vanillin (Table 1, entry 3).

The HZSM5 series of zeolites showed variable activity depending on their SiO_2_/Al_2_O_3_ ratios. With a ratio of 30 (HZSM5‐30), a moderate yield of 36% to BP1 was achieved at 81% conversion of vanillin (Table 1, entry 6). However, when the SiO_2_/Al_2_O_3_ ratio was increased to 50, the conversion decreased to 38%, and the BP1 yield drastically dropped to 9% (Table 1, entry 7). Further increasing the ratio to 80 did not change the BP1 yield beyond 9% (Table 1, entry 8), although the conversion increased from 38% to 45% compared to HZSM5‐50. Nonetheless, the selectivity (≈20%) toward BP1 remained nearly unchanged. Similar to HCZB catalysts, the number of acid sites in HZSM5 zeolites also plays a decisive role in determining their activity and selectivity. In general, for HZSM5 catalysts too, the higher the acidity, the higher the BP1 yield. The highest BP1 yield was observed at a ratio of 30, where the number of acid sites appeared optimal.

For the two commercially available HY zeolites with SiO_2_/Al_2_O_3_ ratios of 5.1 (the lowest among all the zeolites tested) and 60, an opposite trend was observed between the amount of acidity and the BP1 yield. HY‐5.1 provided a low BP1 yield of 8% at 20% conversion (Table 1, entry 9). In contrast, HY‐60 achieved a significantly higher BP1 yield of 66% at 81% conversion, indicating a reverse trend in acidity with BP1 yield (Table 1, entry 10). Perhaps, too many acid sites are detrimental to the reaction.

Over the mordenite (HCZM) zeolites of SiO_2_/Al_2_O_3_‐ratios of 14, 20.6, and 40, the SiO_2_/Al_2_O_3_‐ratio of 40 (HCZM‐40) showed the best catalytic performance. HCZM‐40 led to the highest BP1 yield of 90% among all the zeolites tested with their different SiO_2_/Al_2_O_3_ ratios at almost full conversion of vanillin (Table 1, entry 13). A moderate BP1 yield of 53% was obtained by using HCZM‐14 (Table 1, entry 11), and a further lower yield of 43% was obtained using the HCZM‐20.6 (Table 1, entry 12). A reverse trend in the amount of acid sites with BP1 selectivity is observed over mordenite zeolites. It is important to note that, comparing the catalytic performance of different zeolites‐HCZB, HZSM5, HY, and HCZM is inherently challenging due to their distinct physicochemical properties. These zeolites differ in textural properties, pore size, pore architecture, acid strength, accessibility of acid sites, and SiO_2_/Al_2_O_3_ ratios. Since acidity is intricately linked to these structural parameters, establishing a direct correlation between BP1 yield and acidity alone is not straightforward. Given these complexities, catalyst selection was ultimately based on experimental activity studies, with the most effective zeolite identified as the one delivering the highest BP1 yield.

In addition to zeolites, representative ion exchange resins AmberLyst‐15 and −36 were also used as solid Brønsted acids.^[^ ^21^ ^]^ The use of AmberLyst‐15 resulted in a 68% yield of BP1 at a vanillin conversion of 95% (Table 1, entry 14). With AmberLyst−36 a lower yield of 36% was achieved with a vanillin conversion of 65% (Table 1, entry 15). Although the AmberLyst ion exchange resins confirm the important role of Brønsted acid sites (BAS) in catalyzing the reaction, their BP1 yields are still inferior to those of HCZM‐40. Most importantly, these resins may have a lower thermal stability compared to zeolites, making them rather unsuitable for later recycling via thermal regeneration. While zeolites are thermally robust and can endure calcination temperatures of several hundred degrees Celsius, AmberLyst resins are polymer‐based and begin to degrade at temperatures above ≈120–150 °C. This limits their use in the synthesis of other BPA derivatives that may require temperatures higher than 150 °C. As a result, conventional high‐temperature calcination methods for removing product residues cannot be applied to these resins, making zeolites more suitable candidates for BPA derivatives synthesis.

In the further course, conventional homogeneous Brønsted acids, H_2_SO_4_ and *p*TSA were tested. The use of H_2_SO_4_ as a catalyst resulted in no conversion of vanillin to BP1 although known in literature for acetal synthesis (Table 1, entry 16).^[^ ^22^ ^]^ The cause of this was the highly corrosive nature of H_2_SO_4_, which almost completely carbonized erythritol and was therefore no longer available for the acetal formation. In contrast, a substantial BP1 yield of 78% was obtained by using *p*TSA as a catalyst (Table 1, entry 17). Although these test reactions further confirm the necessity of an appropriate amount and strength of BAS, they are homogeneous acid catalysts and thus encounter separation issues, limiting their application. In comparison, the use of pure Lewis acidic catalysts showed no activity towards BP1 formation. With Al_2_O_3_, no conversion of vanillin was observed (Table 1, entry 18). By using Sc(SO_3_CF_3_)_3_, vanillin could be converted, but no BP1 formation was observed (Table 1, entry 19). These conditional experiments further confirm that BAS are the real active sites for the BP1 synthesis.

All in all, BP1 was successfully synthesized in 5%–90% yield using a variety of homo ‐and heterogeneous catalysts with different properties. The comparison with Lewis and Brønsted acids both homogeneous and heterogeneous leads to the conclusion that only BAS are responsible for the reaction. The highest yield of 90% to BP1 was obtained with the HCZM‐40 zeolite catalyst. Consequently, further research will focus on HCZM zeolites with different SiO_2_/Al_2_O_3_ ratios, with characterization efforts directed at identifying the critical features that influence catalytic activity across these variations. This will help establish structural and compositional parameters to guide the rational design of these catalysts.

### 2.2 Kinetics and Reaction Mechanism

**Figure 1:**
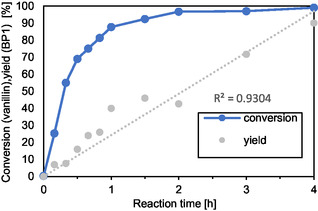
Kinetic experiments of the vanillin conversion to BP1. Reaction conditions: *n* (vanillin) = 3 mmol, *n* (erythritol) = 1.8 mmol, HCZM‐40 = 10 wt%, 25 mL toluene, *T* = 130 °C, and air stream. Conversion and yield are determined by ^1^H‐NMR spectroscopy using 1,3,5‐methoxybenzene as an internal standard.

**Figure 2:**
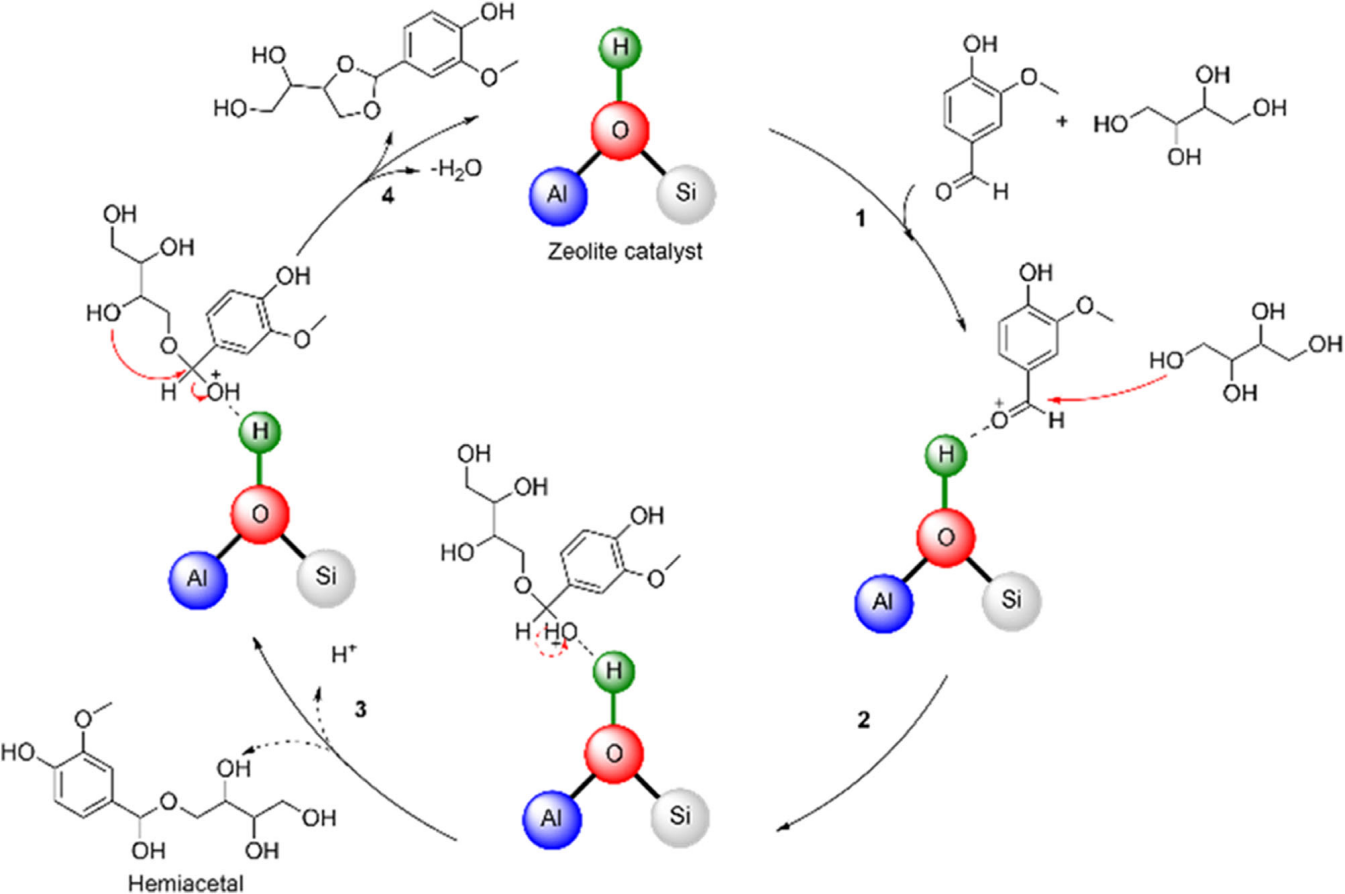
Tentative reaction mechanism for the acetalization of vanillin with erythritol on the HCZM zeolite.

In order to gain a deeper insight into the reaction network and the kinetics of the reaction, a reaction time study was conducted with the best performing catalyst, HCZM‐40. Due to the high boiling points of BP1 and similar polarity of different components involved, nuclear magnetic resonance (NMR) spectroscopy was employed to monitor the reaction. **Figure** **1** reveals that the reaction initially proceeds very rapidly due to the high concentration of substrate and the increased likelihood of these molecules encountering the acid sites on HCZM‐40. As the reaction continues, vanillin conversion reaches a plateau after 2 h. In contrast, the formation of BP1 increases at a much slower, more linear rate, suggesting this step to be the rate‐determining step of the reaction. Notably, within the first 0.5 h, around 70% of the vanillin was converted, yet BP1 yield was only about 16%. This gradual increase in BP1 yield with extended reaction time suggests the formation of intermediate compounds that subsequently react to produce the BP1. This would explain the high conversions obtained with various zeolite catalysts, AmberLysts, and pTSA in Table 1, but the low BP1 yields. **Figure** **2** proposes a plausible reaction mechanism for the acetalization reaction of vanillin with erythritol, based on kinetic studies and the mechanism of acetalization reactions generally known in the literature.^[^ ^23^ ^]^ In the first step, the carbonyl group of vanillin is activated by the H^+^‐site on the HCZM‐40 catalyst (1). The resulting carbenium ion can then be attacked by the electron‐rich ‐OH group of erythritol (2), which leads to the formation of a hemiacetal. This is the fastest step of the reaction. The hemiacetal intermediate can split off from the zeolite's acid site and then react again with vanillin in step 1 either to form a full acetal or a second hemiacetal. It is also possible for the hemiacetal to react in a cyclization by attack of the terminal ‐OH group and subsequent H_2_O removal to form a fully acetalized monophenol acetal. This monophenol acetal then substitutes erythritol in step 1 for hemiacetal formation. The catalytic cycle thus continues until the formation of BP1 is complete making it the rate‐limiting step of the reaction.

### 2.3 Catalyst Recycling

**Figure 3:**
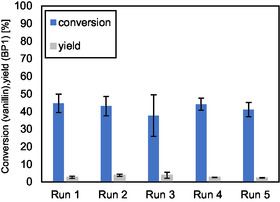
Catalytic recycling experiment of the acetalization reaction of vanillin and erythritol to form BP1. Reaction conditions: *n*(vanillin) = 30 mmol, *n*(erythritol) = 18 mmol, [HCZM‐40] = 10 wt%, 50 mL toluene, *T* = 130 °C, *t* = 20 min, air stream. After each run, the catalyst was washed with ethanol and acetone, then calcined at 550 °C for 6 h.

To evaluate the stability of HCZM‐40, its recyclability was systematically tested. **Figure** **3** shows the catalyst's performance over five consecutive reuse cycles, with intermediate calcination applied before each run. The catalyst maintained consistent activity throughout these cycles. However, without intermediate calcination, a decline in performance was observed from the fourth cycle onward (Figure S1, Supporting Information). Thermogravimetric analysis (TGA) (Figure S2, Supporting Information) confirmed that this loss of activity was due to the accumulation of organic residues on the catalyst and leading to a decrease of available acid sites on the catalyst surface. Therefore, intermediate calcination is an effective regeneration method to maintain catalyst stability during prolonged use. Further recycling experiments showed that washing the catalyst with water led to a rapid decrease in catalytic activity after the second recycling run. These results indicate that water exposure significantly affects both the equilibrium of the reaction and the stability of the HCZM‐40 catalyst (Figure S3, Supporting Information). To confirm this, small amounts of water (65 mg) were added to a reaction solution. After 4 h of reaction time, no conversion to BP1 was observed. The detrimental influence of water during recycling is clearly illustrated in Figure S3, Supporting Information, which depicts the catalytic activity over multiple recycling runs with water washing. However, significant structural changes in the HCZM‐40 have not been observed over the five recycling runs by X‐Ray diffraction (XRD) measurements before and after use (Figure S7, Supporting Information). Moreover, scale‐up experiments were also performed in the 100 g range in order to test the applicability of the reaction under more realistic conditions. For these larger‐scale reactions, the setup was adapted by using an overhead stirrer, which ensured sufficient mixing of the reaction solution as magnetic stirring was no longer adequate at this scale. In these experiments, a yield of 65% BP1 was obtained, confirming that the process can be transferred to a preparative scale.

### 2.4 Influence of Acidity on BP1 Formation: Structure‐Activity Correlations

Given that HCZM‐40 zeolite achieved the highest BP1 yield, a comprehensive investigation of its physicochemical properties is attempted. This in‐depth analysis will provide a better understanding of the factors contributing to its superior catalytic performance. As the catalytic activity of HCZM zeolites is primarily governed by their acid sites, the total acidity of these zeolites was first quantified using temperature‐programmed desorption of ammonia (NH_3_‐TPD) measurements. Figure S4, Supporting Information, shows the NH_3_‐TPD profiles of the catalysts. The TPD profiles show two peaks centered around 150 °C and 450 °C. The peak at 150 °C is due to physisorbed NH_3_, and the one centered at 450 °C is attributed to chemisorbed NH_3_.^[^ ^24^ ^]^ The relative contribution of these acid sites (weak acid sites, between 150 °C and 300 °C, and strong acid sites, above 300 °C) are presented in **Table** **2**.

**Table 2:** Distribution of acid sites in HCZM catalysts deduced from NH_3_‐TPD measurements.

Zeolite	Acid density [mmol g^−1^]		
	Weak acidity	Strong acidity	Total
HCZM‐14	1.02	0.17	1.1
HCZM‐20.6	0.59	0.16	0.74
HCZM‐40	0.52	0.17	0.68

In general, the amount of total acid sites decreases with decreasing aluminum content of HCZMs as anticipated. While HCZM‐14 and HCZM‐20.6 show a slight decrease in BP1 yield (Table 1, entries 11 and 12) with a decreasing acid density, it is contrary to expectations that HCZM‐40 shows the lowest acidity but the highest yield of BP1 (Table 1, entry 13). Possible reasons for this will be analyzed in the following discussion.

**Figure 4:**
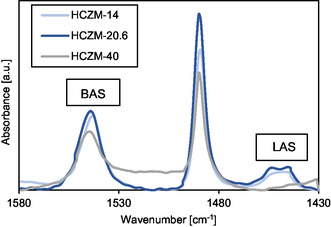
Py‐FTIR spectra of the different HCZM zeolites.

Since it was confirmed in previous experiments with Brønsted‐acidic AmberLyst catalysts (Table 1, entries 14 and 15), as well as with Lewis‐acidic Al_2_O_3_ (Table 1, entry 18) and metal triflates (Table 1, entry 19), that catalytic activity is primarily linked to BAS, an attempt was made to correlate the amount of BAS of HCZMs with the BP1 yield. While NH_3_‐TPD offers the possibility to determine the total acid density, it is not possible to specifically distinguish between BAS and Lewis acid sites (LAS). Therefore, pyridine adsorbed Fourier transform infrared spectroscopy (py‐FTIR) measurements were performed to differentiate and quantify between BAS and LAS. **Figure** **4** shows the py‐FTIR spectra of the HCZM zeolites. The band at 1455 cm^−1^ is attributed to pyridine coordinated to LAS, while the band at 1490 cm^−1^ arises from a combination of interactions, including pyridine bound to both BAS and LAS, as well as hydrogen‐bonded pyridine on the zeolite surface. The band at 1545 cm^−1^ corresponds to pyridinium ions formed by protonation at BAS.^[^ ^25^ ^]^ To accurately quantify the LAS and BAS, these are calculated using the results of the NH_3_‐TPD measurement. The total amount of LAS and BAS can be determined from the relative Lewis/ Brønsted acid ratios (*R*_L/B_) determined by Py‐FTIR. The values for *R*_L/B_ were calculated with the extinction coefficients 2.22 cm μmol^−1^ for Lewis acidity and 5.3 cm μmol^−1^ for Brønsted acidity.^[^ ^25^ ^]^ Furthermore the total amount of acid center sites a_L+B_ determined by NH_3_‐TPD are taken into account.^[^ ^26^ ^]^

(1) $$BAS=\frac{a_{L+B}}{1+R_{L/B}}$$

(2) $$LAS=\frac{a_{L+B}*R_{L/B}}{1+R_{L/B}}$$

The calculated values are shown in **Table** **3**. The results initially exhibit a similar trend in total acidity as observed in the NH_3_‐TPD measurements. As the aluminum content decreases, the total acid sites in the zeolites also decrease. The amount of BAS in HCZM zeolites follows the trend as 1.01 mmol g^−1^ for HCZM‐14, 0.67 mmol g^−1^ for both HCZM‐20.6 and HCZM‐40. Interestingly, the measured amount of BAS did not directly match the BP1 yield. Specifically, HCZM‐40 contains 0.34 mmol g^−1^ fewer BAS than HCZM‐14, yet it achieves a 90% BP1 yield compared to only 50% for HCZM‐14. This discrepancy implies that the BP1 yield is not solely determined by the quantity of BAS on HCZM zeolites.

**Table 3:** Calculated proportions of LAS and BAS using py‐FTIR analysis.

Zeolite	Acid density [mmol g^−1^]			
	BAS	LAS	Total	*R*_L/B_
HCZM‐14	1.01	0.09	1.1	0.09
HCZM‐20.6	0.67	0.07	0.74	0.10
HCZM‐40	0.67	0.003	0.67	0.01

To explore this further, the accessibility of the reactants‐vanillin and erythritol to the BAS was analyzed. Pyridine, which has a kinetic diameter of ≈5.4 Å,^[^ ^27^ ^]^ can easily access the 12‐membered ring (12MR) channels of mordenite (measuring 6.5 × 7.0 Å). However, the BAS located within the smaller 8‐membered ring (8MR) channels (2.6 × 5.7 Å) are less accessible to pyridine. Moreover, based on structural estimations, the kinetic diameters of vanillin and erythritol have been estimated to be about 7‐8 Å and 6‐7 Å, respectively. This suggests that steric hindrance is encountered by these reactants when accessing the acid sites within the 12MR channels, even though pyridine can reach them. Consequently, the correlation of the amount of BAS – as determined by py‐FTIR – with catalytic activity is limited, as the accessibility of the acid sites plays a more significant role than their mere quantity.

As pore diffusion may be limited due to the size of the reactant molecules, the external BAS on HCZM zeolites could play a crucial role in influencing BP1 formation. For this, at first, nitrogen physisorption measurements were carried out to investigate the textural properties of the HCZM zeolites in more detail. The adsorption isotherms (Figure S5, Supporting Information) follow the Langmuir model type 1, which is typical for microporous materials.

**Table** **4** shows that HCZM‐40 has the highest specific surface area of 458 m^2^ g^−1^. The specific surface areas of HCZM‐14 and HCZM‐20.6 are moderately smaller with 405 and 409 m^2^ g^−1^, respectively. Of particular interest are the proportions of external surface area. The external surface area of HCZM‐40 (75 m^2^ g^−1^) is more than double that of HCZM‐14 and HCZM‐20.6 (33 m^2^ g^−1^ for both). Although HCZM‐14 and HCZM‐20.6 have the same external surface area, their BP1 yields differ significantly, with HCZM‐14 showing 53% and HCZM‐20.6 only 43%. This discrepancy can be attributed to the lower number of BAS in HCZM‐20.6, which results from its higher SiO_2_/Al_2_O_3_ ratio compared to HCZM‐14. In contrast, despite having fewer BAS, HCZM‐40's substantially higher external surface area compensates for this, leading to a greater proportion of BAS on the external surface, thus resulting in a higher BP1 yield. Therefore, both the SiO_2_/Al_2_O_3_ ratio (the amount of BAS) and the external surface area are crucial factors for achieving higher BP1 yields with HCZM zeolites.

**Table 4:** Textural properties of HCZM zeolites.

Zeolite	*S*_BET_ [m^2^ g^−1^]	*S*_Ext_ [m^2^ g^−1^]	Pore volume [cc g^−1^]
HCZM‐14	405	33	0.194
HCZM‐20.6	409	33	0.194
HCZM‐40	458	75	0.239

**Figure 5:**
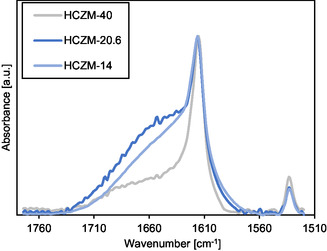
FTIR spectra of adsorbed dTBPy on mordenite surface acidic sites.

In order to draw more accurate conclusions about the available BAS on the external surface of the HCZM catalysts, FTIR measurements were carried out with 2,6‐di‐tert‐butylpyridine (dTBPy) as the probe molecule. Due to its sterically more demanding structure (kinetic diameter = 8 Å),^[^ ^28^ ^]^ this probe molecule can only interact with the BAS on the external surface of the HCZMs and an interaction with the BAS in the internal structure is avoided. In this way, a targeted investigation of the external BAS could be carried out. The amount of chemisorbed dTBPy can be calculated using its extinction coefficient and proper deconvolution of the bands (Figure S6, Supporting Information).^[^ ^27^ ^]^ **Figure** **5** shows the normalized FTIR spectra of the adsorbed dTBPy on HCZM zeolites. The band at about 1615 cm^−1^ can be assigned to dTBPyH^+^, which binds to the BAS on the external surface of HCZMs.^[^ ^27^ ^]^ The quantification results of the dTBPy‐FTIR measurements are presented in **Table** **5**. HCZM‐40 holds the highest amount of external BAS with 120 μmol g^−1^. The external BAS of HCZM‐14 and HCZM‐20.6 amount to 105 and 99 μmol g^−1^. A clear trend can be seen here that the yield of BP1 increases with increasing external BAS. HCZM‐40 has the highest external BAS, which explains the highest BP1 yield.

**Table 5:** External BAS of HCZM zeolites determined by dTBPy‐FTIR analysis.

Zeolite	External BAS [μmol g^−1^]	Accessibility factor
HCZM‐14	105	0.1
HCZM‐20.6	99	0.14
HCZM‐40	120	0.17

In addition, the broad band between 1630 and 1650 cm^−1^ in Figure 5 corresponds to the H‐O‐H bending, which indicates adsorbed water on the catalyst.^[^ ^29^ ^]^ In general, the affinity of the zeolite to adsorb water decreases with decreasing aluminum content.^[^ ^30^ ^]^ The area of this band for HCZM‐20.6 is ≈1.2 times higher than the band of HCZM‐14. Compared to HCZM‐40, the area of this band for HCZM‐20.6 is roughly 2.8 times greater. Adsorbed water from the acetalization reaction can block the active acid sites as well as it could shift the equilibrium of the reaction to the reactant side. As HCZM‐40 has a lower affinity of adsorbing the released water (higher hydrophobicity), this could also provide a favorable effect on the BP1 formation. Our previous study showed that HCZB, HZSM‐5, and HY possess higher external surface areas than HCZM zeolites.^[^ ^24^ ^]^ Assuming these zeolites also exhibit higher external BAS as a result, their lower BP1 yields compared to HCZM‐40 suggest that external BAS alone does not govern catalytic performance. This emphasizes the role of additional structural and physicochemical factors, making direct comparisons across different zeolites inherently complex, as previously discussed.

**Figure 6:**
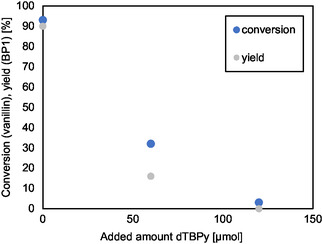
Results of the catalytic reaction of vanillin and erythritol to BP1 with completely and partially blocked external BAS by dTBPy on the HCZM‐40 surface.

To further confirm the influence of the external BAS on BP1 yield, the surface BAS were selectively blocked and deactivated with dTBPy before the reaction. For this purpose, HCZM‐40 was added to a flask containing toluene, and specific amounts of dTBPy were introduced to block half or all of the external BAS (the values deduced from dTBPy‐FTIR, Table 5). The results in **Figure** **6** show that with decreasing external BAS availability, the conversion of vanillin as well as the BP1 yield decreases. This is an additional indication that only external BAS are involved in the reaction, avoiding pore‐limitations in catalytic activity. The accessibility factor (AF) can also be taken into consideration. This is defined by the ratio of external BAS and the total number of BAS, determined by py‐FTIR measurements.^[^ ^27^ ^]^ This shows that HCZM‐40 also exhibits the highest AF value of 0.17 (Table 5) further validating the role of external BAS.

## 3 Conclusion

This study successfully demonstrates the sustainable synthesis of BPA alternatives through the acetalization of renewable vanillin and erythritol, using zeolites as heterogeneous catalysts. A comprehensive catalyst screening was conducted, evaluating industrially relevant zeolites (Hβ, ZSM5, HY, and MOR) with different SiO_2_/Al_2_O_3_ ratios, Brønsted acidic resins, homogeneous acids such as H_2_SO_4_ and pTSA, as well as Lewis acidic catalysts including heterogeneous Al_2_O_3_ and homogeneous Sc(SO_3_CF_3_)_3_. The results confirmed that BAS are essential for the reaction, while LAS remained inactive. Among all tested catalysts, mordenite zeolite with SiO_2_/Al_2_O_3_ ratio of 40 (HCZM‐40) exhibited the highest activity, achieving a 90% BP1 yield at complete vanillin conversion.

The kinetic studies indicated a two‐step mechanism for the acetalization reaction, characterized by an initial fast step followed by a slower, rate‐limiting step. This behavior is consistent with mechanisms reported in the literature, which propose the rapid formation of a hemiacetal intermediate preceding a slower conversion to the acetal. Based on this correlation, we hypothesize that the reaction proceeds via a hemiacetal intermediate, with its transformation to the acetal constituting the rate‐limiting step. Catalyst recyclability study showed that HCZM‐40 experienced deactivation after three consecutive cycles due to carbon deposition; however, activity was fully restored with an intermediate calcination step, enabling stable performance over five consecutive runs. The structure‐activity correlation study of HCZM zeolites with varying SiO_2_/Al_2_O_3_ ratios revealed that total acidity had no direct influence on BP1 yield. Likewise, the total concentration of BAS, as measured by py‐FTIR, showed no clear relationship with catalytic performance. However, a detailed reactant accessibility analysis and study using dTPBy as probe via FTIR, which selectively titrates external BAS, combined with textural analysis, established that only the BAS on the external surface are accessible and active for the acetalization reaction. HCZM‐40 outperformed other mordenites (HCZM‐20.6 and HCZM‐14) due to its higher concentration of external BAS and larger external surface area. Additionally, selective poisoning experiments with dTPBy further validated the exclusive role of external BAS in the acetalization reaction. Moreover, dTPBy‐FTIR analysis indicated that HCZM‐40 is more hydrophobic than other catalysts, which also contributed to its superior catalytic performance.

In conclusion, this study not only demonstrates the potential of zeolites as effective catalysts for the sustainable synthesis of BPA alternatives but also provides valuable insights into catalyst design, emphasizing the critical role of external BAS accessibility and surface hydrophobicity in optimizing zeolite catalysts for BPA derivatives synthesis. By utilizing renewable feedstocks and industrially relevant, widely used heterogeneous catalysts, this work advances sustainable chemistry practices and offers a greener approach to BPA derivative production, contributing to more environmentally friendly industrial practices. In addition, the catalyst can be recycled, further enhancing the sustainability of the process. The successful scale‐up experiments highlight the potential for industrial application, demonstrating that the methodology is not only effective on a laboratory scale but also transferable to larger, more practical settings.

## 4 Experimental Section

4.1

4.1.1

##### Chemicals and Materials

Vanillin (99%) was supplied by Roth. Erythritol (99%) and Sc(SO_3_CF_3_)_3_ were supplied by Fluorochem Al_2_O_3_, 1,3,5‐trimethoxybenzene (>99%). AmberLyst −15 and −36 were supplied by Sigma‐Aldrich. Toluene (99.8%), DMSO (99.8%), pyridine (99%), and H_2_SO_4_ (98%) were supplied by Chemsolute. HCZM‐14, −20.6, and −40 and HCZB‐25, −30, and −150 zeolites were provided by Clariant. The ZSM‐5 and Y zeolites were provided by Alfa Aesar in its ammonium form. All zeolites have been calcined at 550 °C for 6 h with a rate of 10 K min^−1^ prior to use. DMSO‐d6 (99.8%) was supplied by Deutero. pTSA monohydrate (98%) was supplied by Bernd Kraft. 2,6‐Ditertbutylpyridin (97%) was provided by abcr.

##### Catalytic Reaction

For the catalytic experiments, a mixture of vanillin (3 mmol), erythritol (1.8 mmol), and 10 wt% catalyst w. r. t vanillin was heated in 25 mL of toluene at 130 °C under reflux for 4 h in a two‐necked round‐bottom flask. A continuous flow of air above the reaction solution and a Dean Stark apparatus were used to continuously remove the water formed during the reaction. After 30–60 min, a fine crystalline colorless precipitate (BP1) was formed. For the screening experiments, at the end of the reaction time, the solvent was removed using a rotary evaporator. Afterward, the crude product was weighed. A sample with addition of specific amount of mesitylene as an internal standard was taken and dissolved in DMSO d6 for quantification via ^1^H‐NMR. For the kinetic, recycling and catalyst poisoning experiments, 1,3,5‐trimethoxybenze was added directly to the reaction solution, and then samples were taken directly from the reaction solution and quantified by ^1^H‐NMR spectroscopy, with DMSO‐d6 as a solvent. The ^1^H‐NMR spectrum for a crude product mixture is shown in Figure S8, Supporting Information. In addition, BP1 can be isolated by reprecipitation. Therefore, the reaction solvent toluene was removed by a rotary evaporator. The remaining residue was dissolved in DMSO and the catalyst was filtered off. By adding distilled water, the product was precipitated out of the DMSO solution and obtained as a fine crystalline colorless solid. The precipitate was filtered off and washed with ethanol and acetone, afterwards the product was dried at 80 °C in a vacuum drying oven giving an isolated yield of 70%.

##### Product Characterization and Quantification

The product was analyzed and quantified by ^1^H NMR analysis. The measurements were performed at room temperature on a Bruker AS 400 spectrometer with DMSO‐d6 as the solvent and mesitylene or 1,3,5‐trimethoxybenzene as the internal standard. A representative ^1^H‐NMR of BP1 is shown in Figure S9, Supporting Information. The multiplicities are denoted as s (singlet), br (broad singlet), d (doublet), t (triplet), q (quartet), quin (quintet), sext (sextet), sep (septet), m (multiplet), dd (doublet of doublets), and ddd (doublet of doublet of doublets). ^1^H‐ NMR spectra were referenced to the residual solvent signal.

^**1**^ **H NMR** (400 MHz, DMSO‐*d*_6_) *δ*: 9.11 (s, 2 H, H‐1), 6.95–6.92 (m, 2 H, H‐5), 6.86–6.82 (m, 2 H, H‐3), 6.75 (d, *J* = 8.1 Hz, 2 H, H‐4), 5.65 (s, 2 H, H‐6), 4.21 (dd, *J* = 9.0, 2.5 Hz, 2 H, H‐8), 3.85 (q, *J* = 9.5 Hz, 4 H, H‐7), 3.76 (s, 6 H, H‐2).

For quantification, the proton of the aldehyde group of vanillin 10.26 (s, 1 H) was used to calculate the conversion. The protons 5.65 (s, 2 H, H‐6) of BP1 were used for the yield calculation. The areas are converted to number of moles. The conversion (X), yield (Y), and selectivity (S) were then calculated based on the following equations.

(3) $$X\left(vanillin\right)=\frac{n_{0}\left(vanillin\right)-n_{t}\left(vanillin\right)}{n_{0}\left(vanillin\right)}$$

(4) $$Y\left(BP1\right)=\frac{n_{t}\left(BP1\right)}{n_{max}}$$

(5) $$S\left(BP1\right)=\frac{Y\left(BP1\right)}{X\left(vanillin\right)}$$

where *n*_0_ is the starting concentration of vanillin, *n*_t_ is the end concentration of the specific molecule, and *n*_*max*_ is the is the maximum possible substance quantity.

##### Catalyst Recycling

For the catalyst recycling experiments, 120 times the amount of substrate and catalyst was used as in the catalytic reaction procedure and 50 mL of toluene as a solvent. The recycling experiments were conducted at 130 °C for 20 min reaction time. For the first recycle run, the reaction solution was filtered off after the end of the reaction time. The filtrate was washed three times with ethanol and acetone and afterward dried at 80 °C for 1 h in a vacuum drying oven. For the second recycle run, prior to use, the catalyst was additionally calcined at 550 °C for 5 h.

##### Catalyst Characterization: N_2_‐Physisorption

The N_2_ adsorption and desorption isotherms of the zeolites were analyzed with a Quadrasorb SI (3 P Instruments) at −196 °C. Before measurement, the samples underwent degassing under vacuum using a FloVac Degasser at 250 °C for 8 h. The Brunauer–Emmett–Teller (BET) method was employed to determine the specific surface area within a relative pressure range of p/p_0_ = 0.05–0.2. The total pore volume was calculated based on the nitrogen adsorption at p/p_0_ = 0.95–0.98. Micropore surface area and volume were assessed through t‐plot analysis.

##### Catalyst Characterization: NH_3_‐TPD Measurements

The analysis was conducted on an AutoChem HP Chemisorption Analyzer (micromeritics). The samples were heated first at 10 °C min^−1^ to 650 °C while being purged with He. The temperature was maintained for 15 min to ensure the removal of surface impurities. Afterward, the device was cooled down to 60 °C and NH_3_ (10% in He) was dispensed onto the samples at a flow rate of 10 mL min^−1^ and heated to 120 °C and held for an additional 20 min. The spectra were recorded from 50 °C while heating the samples to 650 °C at a heating rate of 10 °C min^−1^. Prior to measurement, the samples were purged with He for 60 min and cooled to room temperature.

##### Catalyst Characterization: Py‐FTIR Measurements

Py‐FTIR measurements were performed on a Vertex 70 spectrometer equipped with a transmission cell and an MCT detector (2 cm^−1^ resolution). The zeolite was pressed into a self‐supporting disk using a pellet press. The disk was 10 mm in diameter, containing 25–30 mg of sample. First, the background spectrum of the empty evacuated sample cell was measured at 80 °C. The sample was then heated to 250 °C (at 5 °C min^−1^) for 1 h under vacuum to remove any residues and adsorbed water. Afterwards, the background spectrum of the sample was recorded at 80 °C. In the following, pyridine adsorption was induced by exposing the evacuated sample to pyridine vapor for 2 min. Physisorbed pyridine was removed by treating the sample at 200 °C (at 5 °C min^−1^) for 1 h. Afterwards, the measurement of chemisorbed pyridine was performed at 80 °C.

##### Catalyst Characterization: dTPBy‐FTIR Measurements

The dTBPy‐FTIR‐measurements were performed on the same spectrometer used for py‐FTIR with the same mode of sample preparation. Before measurement, the pellet was placed in a vacuum drying oven overnight at 80 °C. To adsorb dTBPy, the pellet was soaked in 10 mL of dTBPy for 30 min, followed by vacuum drying for another 30 min at 80 °C. The background spectrum of the evacuated empty cell was recorded at 80 °C. The pellet was then placed in the FTIR cell, and the physically adsorbed dTBPy was removed by heating the sample under vacuum at 150 °C for 30 min. Finally, the measurement of chemisorbed dTBPy was recorded at 80 °C.

##### Catalyst Characterization: Catalyst Poisoning with 2,6‐dTBPy

For the controlled catalyst poisoning test, a stock solution of dTBPy was first prepared in toluene (0.012 M). The desired amount of dTBPy was then added to a suspension of the catalyst and toluene and stirred for 60 min at room temperature. Vanillin and erythritol were then added to this suspension and the catalytic reaction was carried out as described earlier.

##### Catalyst Characterization: XRD Measurements

The crystal structure of the HCZM zeolites was examined on a diffractometer D 5000 (Siemens) with Cu Kα radiation (λ = 0.154056 nm 45 kV 40 mA). The diffraction patterns were recorded in the 6–90 2Θ range, with 0.02 intervals and a step time of 1 s.

## Conflict of Interest

The authors declare no conflict of interest.

## Supporting information

Supplementary Material

## Data Availability

The data that support the findings of this study are available at https://doi.org/10.5281/zenodo.15975506.
