# Supplementary material for: Sustainable Bisphenol A Alternatives from Vanillin and Erythritol Using Zeolite Catalysts
Source: ChemSusChem. 2025 Jul 31;18(19):e202500923. doi: 10.1002/cssc.202500923 (PMC12487737; doi:10.1002/cssc.202500923)
Supplement: Supplementary file 1 — Supplementary Material [file CSSC-18-e202500923-s001.pdf]

ChemSusChem

Supporting Information

# Sustainable Bisphenol A Alternatives from Vanillin and Erythritol Using Zeolite Catalysts

Kevin M. Sabel, Joby Sebastian, Regina Palkovits

**Table of contents**

**Figure S1.** ........ 3
**Figure S2.** ........ 3
**Figure S3.** ........ 4
**Figure S4.** ........ 5
**Figure S5.** ........ 5
**Figure S6.** ........ 6
**Figure S7.** ........ 6
**Figure S8** ........ 7
**Figure S9** ........ 7

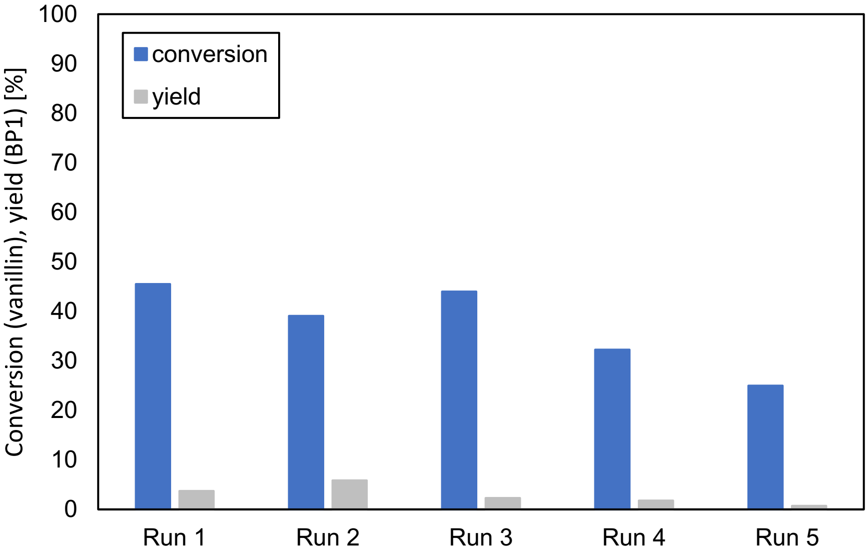


**Figure S1.** Catalytic recycling experiment of the acetalization reaction of vanillin and erythritol to form BP1 showing catalyst deactivation after 3rd run. Reaction conditions: n(vanillin) = 30 mmol, n(erythritol) = 18 mmol, [HCZM-40] = 10 *wt%*, 50 mL toluene, T = 130 °C, t = 20 min, air stream. After each run, the catalyst was washed with ethanol and acetone, dried, and used for the next run.

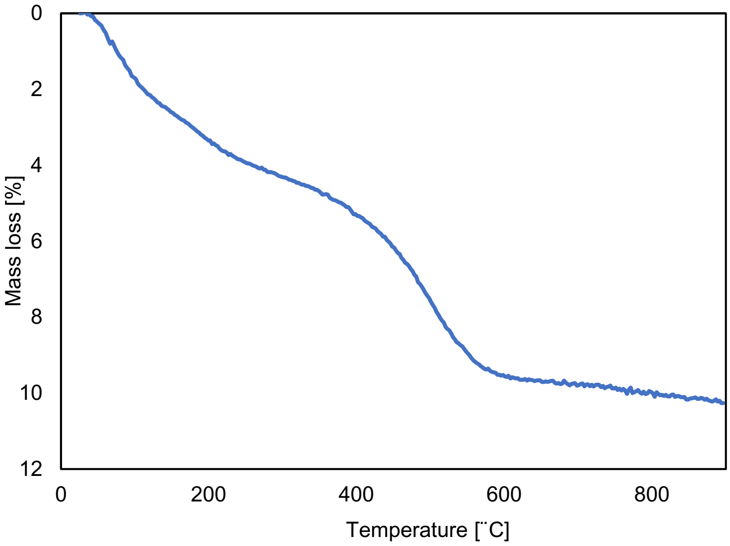


**Figure S2.** TGA analysis of spent catalyst HCZM-40. The profile shows two mass loss points at about 180 °C, which could be due to organic substrate residues, and at about 450 °C, which corresponds to the decomposition temperature of BP1 with a mass loss of about 4%.

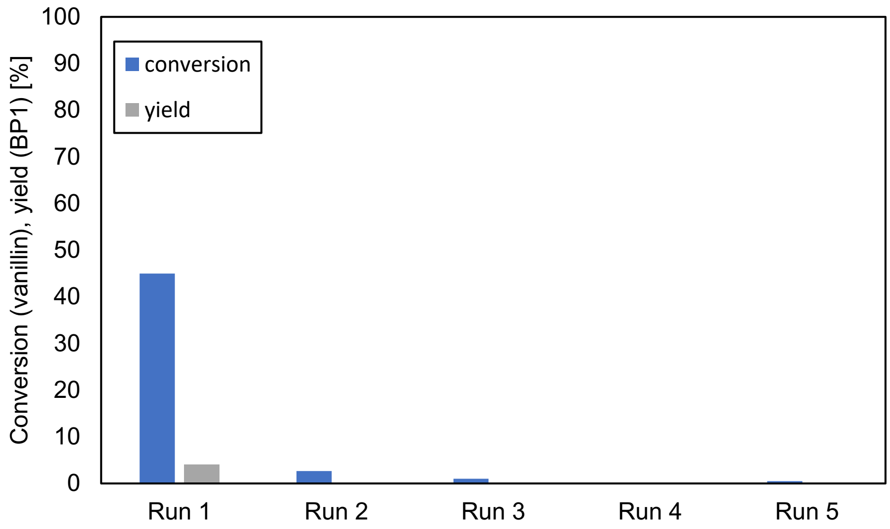


**Figure S3.** Catalytic recycling experiment of the acetalization reaction of vanillin and erythritol to form BP1 showing catalyst deactivation after 2nd run. Reaction conditions: n(vanillin) = 30 mmol, n(erythritol) = 18 mmol, [HCZM-40] = 10 wt%, 50 mL toluene, T = 130 °C, t = 20 min, air stream. After each run, the catalyst was washed with water, ethanol and acetone, dried, and used for the next run

.

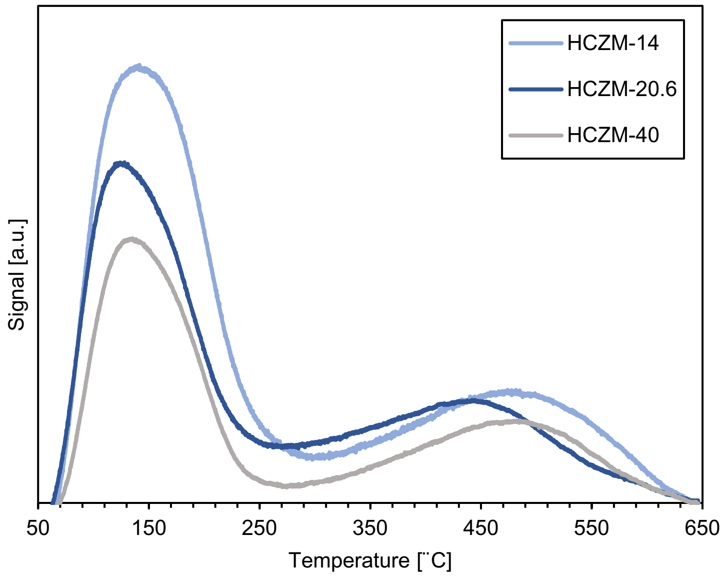


**Figure S4.** $NH_3$-TPD profiles of applied HCZM zeolites with varying $SiO_2/Al_2O_3$ ratio.

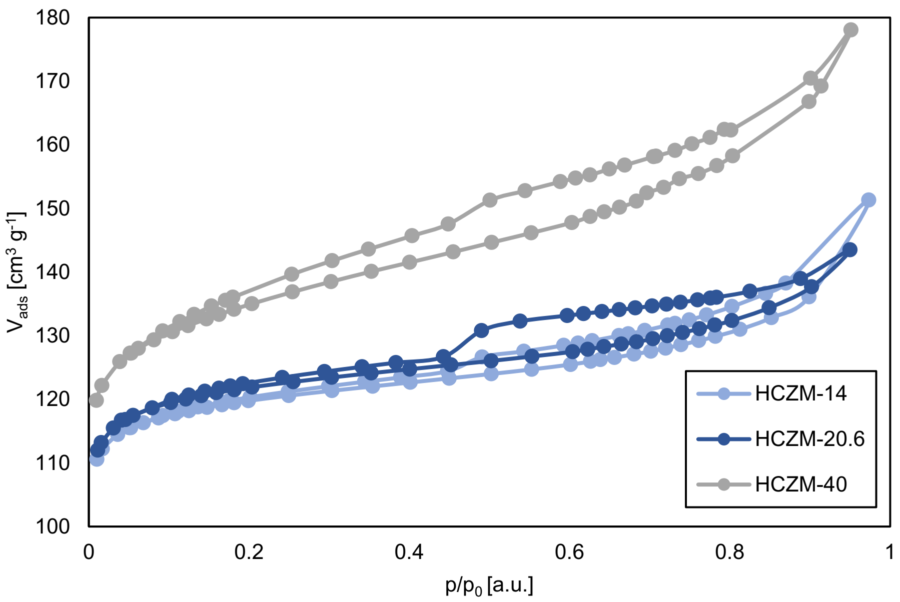


**Figure S5.** $N_2$ physisorption isotherms of the HCZM zeolites with varying $SiO_2/Al_2O_3$ ratios. The adsorption isotherms follow the Langmuir model type 1.

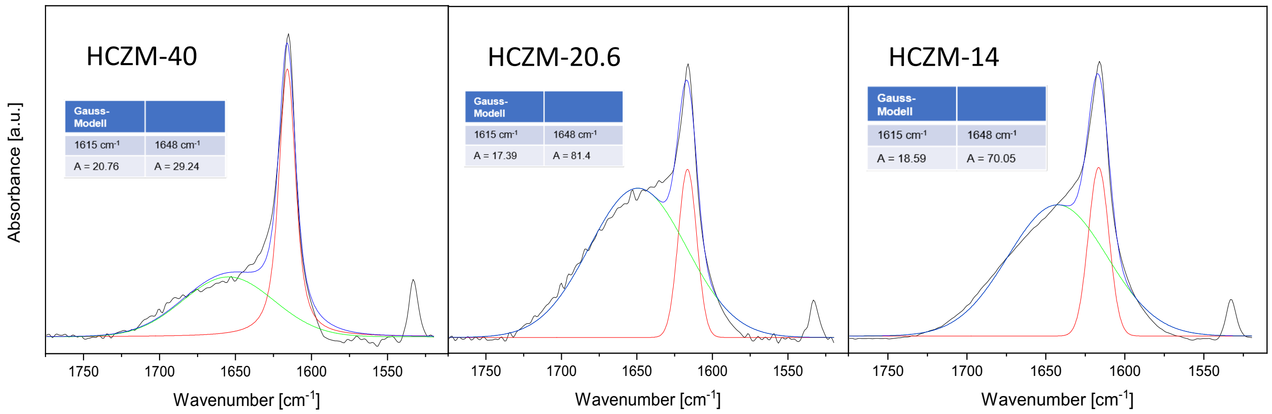


**Figure S6**. Deconvoluted 2,6-dTBPy-FTIR profiles of tested mordenite zeolites.

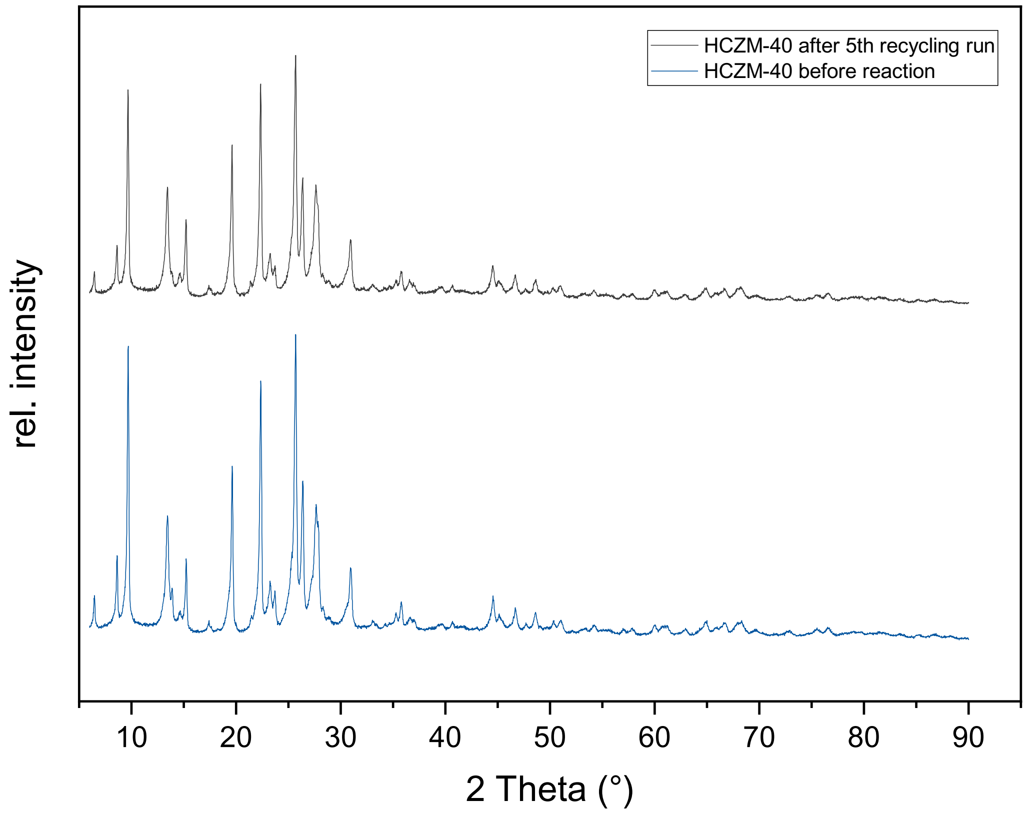


**Figure S7.** X-Ray diffractograms of HCZM-40 before and after 5[th] recycling run, showing no structural changes or decomposition of crystal structure.

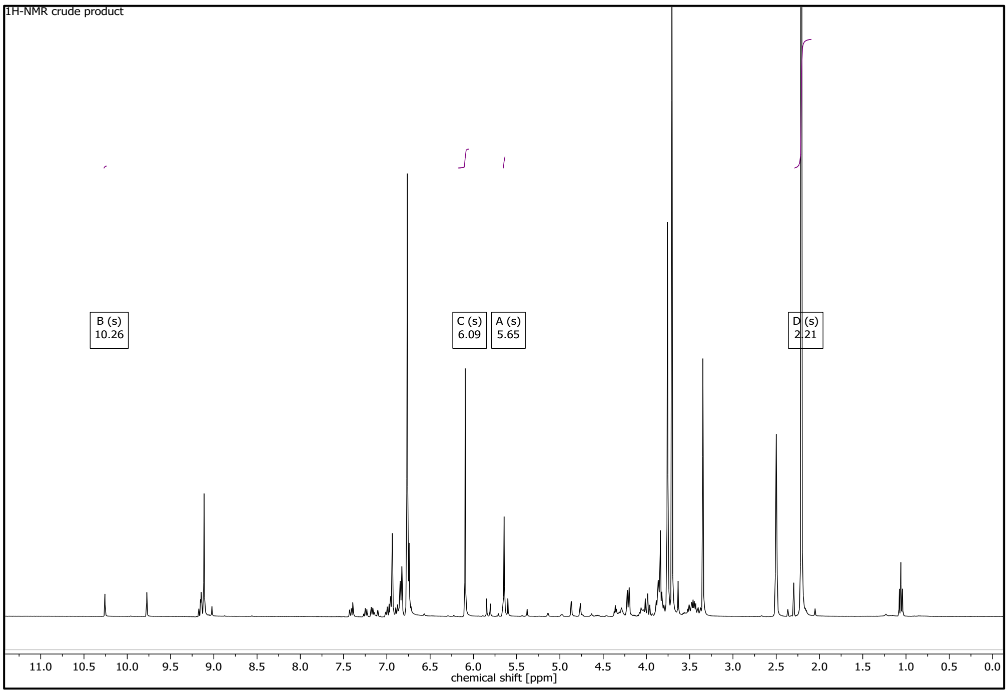


**Figure S8**. Representative $^{1}$H-NMR spectra of crude product mixture. (400 MHz, DMSO-d6). Peak A and B are assigned to BP1 and the vanillin, respectively. The reference peaks of the internal standards 1,3,5-trimethoxybenzene (C) and mesitylene (D) used in each case are also shown as examples.

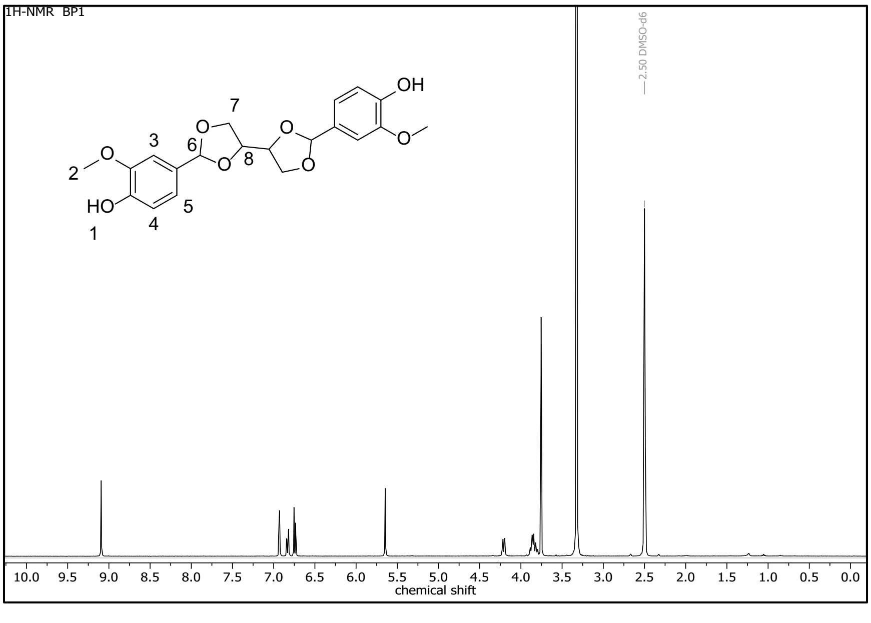


**Figure S9**. $^{1}$H-NMR spectra of purified BP1. (400 MHz, DMSO-$d_6$):δ 9.11 (s, 2H, H-1), 6.95 – 6.92 (m, 2H, H-5), 6.86 – 6.82 (m, 2H, H-3), 6.75 (d, J = 8.1 Hz, 2H, H-4), 5.65 (s, 2H, H-6), 4.21 (dd, J = 9.0, 2.5 Hz, 2H, H-8), 3.85 (q, J = 9.5 Hz, 4H, H-7), 3.76 (s, 6H, H-2).
